# Altered Functional Connectivity of Insular Subregions in Type 2 Diabetes Mellitus

**DOI:** 10.3389/fnins.2021.676624

**Published:** 2021-06-16

**Authors:** Dongsheng Zhang, Man Wang, Jie Gao, Yang Huang, Fei Qi, Yumeng Lei, Kai Ai, Xuejiao Yan, Miao Cheng, Yu Su, Xiaoyan Lei, Xiaoling Zhang

**Affiliations:** ^1^Department of MRI, Shaanxi Provincial People’s Hospital, Xi’an, China; ^2^Department of Graduate, Xi’an Medical University, Xi’an, China; ^3^Department of Clinical Science, Philips Healthcare, Xi’an, China

**Keywords:** type 2 diabetes mellitus, resting-state functional magnetic resonance imaging, functional connectivity, insula, subregion, neuroimaging

## Abstract

Diabetes-related brain damage can lead to cognitive decline and increase the risk of depression, but the neuropathological mechanism of this phenomenon remains unclear. Different insular subregions have obvious functional heterogeneity, which is related to many aspects of type 2 diabetes mellitus (T2DM)-related brain damage. However, little is known about changes in functional connectivity (FC) in insular subregions in patients with T2DM. Therefore, we aimed to investigate FC between different insular subregions and clinical/cognitive variables in patients with T2DM. Fifty-seven patients with T2DM and 55 healthy controls (HCs) underwent a neuropsychological assessment and resting-state FC examination. We defined three insular subregions, including the bilateral dorsal anterior insula (dAI), bilateral ventral anterior insula (vAI), and bilateral posterior insula (PI). We examined differences in FC between insular subregions and the whole brain in patients with T2DM compared with HCs. A correlation analysis was performed to examine the relationship between FC and clinical/cognitive variables. Compared with HCs, patients with T2DM showed significantly decreased FC between the dAI and the right inferior frontal gyrus, right superior/middle temporal gyrus, right hippocampus, and right precentral gyrus. FC between the vAI and the right supramarginal gyrus, as well as the PI and the right precentral/postcentral gyrus, was reduced in the T2DM group compared with the control group. In the T2DM group, we showed a significant negative correlation between glycated hemoglobin concentration and FC in the dAI and right hippocampus (*r* = −0.428, *P* = 0.001) after Bonferroni correction. We conclude that different insular subregions present distinct FC patterns with functional regions and that abnormal FC in these insular subregions may affect cognitive, emotional, and sensorimotor functions in patients with T2DM.

## Introduction

Type 2 diabetes mellitus (T2DM) is a chronic metabolic disease characterized by insulin resistance and long-term hyperglycemia. The number of patients with T2DM worldwide is approximately 445 million, and the incidence is increasing; thus, T2DM is a serious public health problem (Cho et al., 2018). With T2DM progression, patients experience various complications, including cardiopathy, nephropathy, and peripheral neuropathy, as well as diabetic encephalopathy (Sima, 2010). Not only does T2DM-related brain damage cause a decline in various cognitive functions, such as memory, execution, and attention (Macpherson et al., 2017; Cho et al., 2018), but it is also related to abnormal emotion and increases the risk of depression (Stuart and Baune, 2012). A recent study (Lin et al., 2018) showed that functional connectivity (FC) disorder between brain regions is the neural basis of cognitive impairment, and it is also closely related to emotional abnormalities (Price and Duman, 2020). Although previous studies (Chen et al., 2015; Sun et al., 2018; Xia et al., 2018; Tan et al., 2019) have explored the relationship between functional disconnection in the posterior cingulate, hippocampus, thalamus, and amygdala and abnormal cognitive function and emotion in patients with T2DM, the neuropathological mechanism of T2DM-related brain damage remains unclear.

The insula is a key node of the brain network. It plays a role in cognitive and emotional processing and coordinates interactions between large-scale neurocognitive networks (Uddin, 2015). It has attracted much attention in recent years. Previous studies have confirmed that disconnected insular function may be involved in the pathogenesis and development of many neuropsychiatric diseases, including mild cognitive impairment and depression (Xie et al., 2012; Zu et al., 2019). Recent neuroimaging studies on patients with T2DM found that insular neuronal activity is abnormal, and long-range connections are reduced (Zhou et al., 2014; Peng et al., 2016; Liu et al., 2017). Cui et al. (2016) found that the degree centrality of the insula in patients with T2DM is increased, and they speculated that increased FC between the insula and the anterior cingulate gyrus may be related to better executive function. A 5-year longitudinal study (Zhang et al., 2021) confirmed that the insula is a brain region with severely decreased neurovascular coupling in patients with T2DM, which is related to a decline in memory. However, other studies (Roy et al., 2020; Zhang et al., 2020) suggest that decreased insular gray matter volume and abnormal neuronal activity are significantly related to negative emotions (anxiety and depression) in patients with T2DM. In addition, Li et al. (2018) found that under temperature stimulation, the insula and other sensorimotor regions were activated in patients with T2DM with peripheral neuropathy, which suggests that the insula may also play a role in sensorimotor impairment. Although these studies show that insular dysfunction and FC disorder are closely related to the neural mechanism of T2DM-related brain damage, the above results are heterogeneous, and it is difficult to determine the specific role of the insula in T2DM-related brain damage. This may be because almost all previous neuroimaging studies that have investigated insular connectivity in T2DM have treated the insula as a single, homogeneous region, while ignoring functional heterogeneity of insular subregions.

In recent years, neuroimaging research (Deen et al., 2011) has divided the insula into the dorsal anterior insula (dAI), the ventral anterior insula (vAI), and the posterior insula (PI) according to the structural connections and functional characteristics of the insula. It has also been confirmed that different subregions have distinct FC patterns. The dAI participates in higher-level cognitive processes, and the vAI is involved in emotional processing (Chang et al., 2013; Uddin et al., 2014). The PI is associated with motor-related functions and sensory perception (Mutschler et al., 2009; Kurth et al., 2010). Recently, Liu et al. (2018) found that different insular subregions present distinct resting-state FC patterns with various functional networks that are affected by Alzheimer’s disease (AD). Not only does T2DM have a similar neuropathological mechanism to AD (Bedse et al., 2015), but the functions of different subregions of the insula are also closely related to many aspects of T2DM-related brain damage. However, as far as we know, no studies have explored the FC pattern of insular subregions in patients with T2DM. Considering significant functional heterogeneity in different insular subregions, it is necessary to explore the role of different insular subregions in the context of T2DM-induced brain damage.

Seed-based FC analysis often reveals FC in specific brain regions according to prior anatomical knowledge (Lowe et al., 2002), so it can be used to identify a set of core networks with highly correlated activities. It has become an important method to explore the neural mechanisms of neuropsychiatric disease. This study aimed to use the resting-state FC method to explore FC between different insular subregions (dAI, vAI, and PI) and the whole brain in patients with T2DM. We speculate that different subregions of the insula in patients with T2DM have distinct FC patterns. Abnormal FC in the dAI, vAI, and PI may be related to different aspects of T2DM-related brain damage.

## Materials and Methods

### Study Subjects

Sixty patients with T2DM and 57 healthy controls (HCs) were recruited for this study from June 2019 to October 2020. Patients were recruited from the Department of Endocrinology, Shaanxi Provincial People’s Hospital, while HCs were recruited via advertisements.

The inclusion criteria were as follows: (1) >40 years of age, right-handed, and at least 6 years of education; (2) diagnosis of T2DM according to the 2014 criteria of the American Diabetes Association; and (3) fasting blood glucose (FBG) concentration <6.1 mmol/L and no symptoms of diabetes (HCs only). Exclusion criteria for all subjects included the following: (1) conventional brain magnetic resonance imaging (MRI)-identified central nervous system disease (e.g., cerebrovascular accident, tumor, and infection); (2) psychiatric or neurological disorders that could influence cognitive function; (3) major medical conditions, such as anemia, cancer, or thyroid dysfunction; (4) a history of stroke or alcohol/drug dependence; (5) a Mini-Mental State Examination (MMSE) score of <24; and (6) contraindications to MRI. In addition, subjects were excluded from the study if they had a history of hyperglycemia episodes (blood glucose > 33.3 mmol/L) or severe hypoglycemia episodes (blood glucose < 3.9 mmol/L). Every subject arrived at the department for MRI at 6:30–7:00 p.m. after dinner and controlled their blood glucose according to their doctor’s orders on the day of the scan. MRI was performed after approximately 30 min of structured clinical interview and a series of psychological tests. Only one subject was scanned each day to ensure that each subject completed the examination with relatively stable blood glucose. The test procedure and scan time of HCs were the same as those of patients with T2DM. All subjects were awake during the scan and did not experience discomfort. The study was reviewed and approved by the Ethics Committee of Shaanxi Provincial People’s Hospital. The study protocol was explained to all subjects, and all subjects provided written informed consent to participate.

### Demographic and Clinical Data

Demographic and clinical data of all subjects were collected from medical records and questionnaires, including education level, smoking status, blood pressure, body mass index (BMI), and disease duration (for subjects with T2DM only). Blood samples were obtained by venipuncture at 8 a.m. after overnight fasting for at least 8 h, to determine FBG, glycated hemoglobin (HbA1c), and blood lipid levels.

### Neuropsychological Tests

All subjects underwent a neuropsychological examination covering multiple cognitive domains. The MMSE and the Montreal Cognitive Assessment (MoCA) were used to assess general cognitive function. Trail-Making Test A (TMT-A) was used to evaluate neural response speed and attention, while visual memory and visuospatial ability were measured using the Clock-Drawing Test (CDT). The Beck Depression Inventory (BDI) was used to assess the presence of depression in patients.

### MRI Acquisition

All MRI images were acquired using a 3.0-T MRI scanner (Ingenia, Philips Healthcare, Netherlands) with a 16-channel phased-array head coil. All subjects were instructed to lie down quietly, keep their eyes closed without falling asleep, relax their minds, and avoid thinking of anything during scanning. Pads were placed on both sides of the coil to reduce artifacts caused by head movement. Earplugs were used to reduce the noise of the scanner. Conventional axial T1-weighted, T2-weighted, and T2 fluid-attenuated inversion recovery (FLAIR) sequences were performed to exclude visible brain disease. Resting-state functional MRI (fMRI) data were obtained using a gradient-echo planar sequence with the following parameters: repetition time (TR) = 2,000 ms, echo time (TE) = 30 ms, number of slices = 34, thickness = 4 mm, gap = 0 mm, field of view (FOV) = 230 mm × 230 mm, acquisition matrix = 128 × 128, flip angle (FA) = 90°, and 200 volumes. Sagittal three-dimensional T1-weighted images were acquired with the following parameters: TR = 7.5 ms, TE = 3.5 ms, FA = 8°, FOV = 250 mm × 250 mm, acquisition matrix = 256 × 256, slice thickness = 1 mm, no gap, and 328 sagittal slices.

### Imaging Data Analysis

Preprocessing of fMRI data was performed using the programs in Data Processing & Analysis for Brain Imaging v3.0 (DPABI: http://rfmri.org/dpabi), which is based on Statistical Parametric Mapping (SPM12, http://www.fil.ion.ucl.ac.uk/spm). The first 10 volumes of fMRI images were removed to avoid heterogeneity in the initial MRI signal. The remaining volumes were preprocessed according to the following steps. Slice timing and realignment were performed to correct for head motion. Any subjects with a head motion >1.5 mm of translation or a 1.5° rotation in any direction were excluded. Volumes were spatially normalized to the standard Montreal Neurological Institute space and resampled into 3 × 3 × 3-mm^3^ voxels. Multiple regression models were used to remove the effects of covariates of no interest, which involved 24 motion parameters, cerebrospinal fluid signals, and white matter signals. The obtained images were smoothed using a Gaussian filter of 6-mm full width at half-maximum. Then, detrending and filtering (0.01–0.08 Hz) were performed.

**FIGURE 1:**
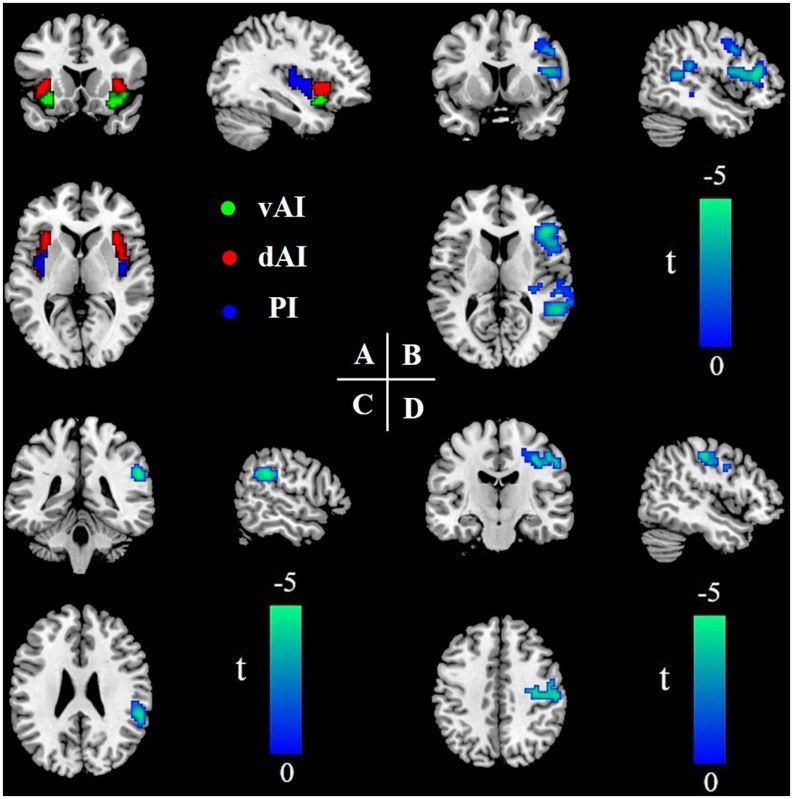
Insular subregions and differences in resting-state FC between the T2DM group and the HC group (two-sample *t*-test, *P* < 0.05, GRF-corrected). **(A)** Bilateral insular subregions, including the dorsal anterior insula (dAI; red), ventral anterior insula (vAI; green), and posterior insula (PI; blue). **(B)** Reduced FC of the dAI in T2DM. **(C)** Reduced FC of the vAI in T2DM. **(D)** Reduced FC of the PI in T2DM. L, left; R, right; FC, functional connectivity; T2DM, type 2 diabetes mellitus; HC, healthy control; and GRF, Gaussian random field.

We parcellated the insula in both hemispheres into three subregions based on a previous study (Deen et al., 2011). First, the region of interest (ROI) of the insula was extracted according to the automated anatomical labeling standard brain. Second, Pearson’s correlation was calculated to create FC maps between the insular region and the whole brain. Then, we applied Fisher’s *r*-to-*z* transformation to FC maps of each subject. Third, the *k*-means clustering algorithm was used in connectivity maps by using the squared Euclidean distance as the distance measure. The *k*-means clustering was repeated 300 times, and the solution with the minimal within-cluster variance was selected. Finally, we defined *k* = 3 to parcellate the insula into three distinct subregions, i.e., the vAI, dAI, and PI. The same subregions of the insula in both hemispheres were combined as one ROI, i.e., dAI, vAI, and PI (Figure 1A).

For each subject, the mean blood oxygen level-dependent time course of each ROI was extracted, and time correlation coefficients between each ROI and other brain regions were calculated. Correlation coefficients were converted to *Z*-values using the Fisher *r*-to-*z* transformation to improve normality. *Z*-values represent the strength of FC between the voxel and ROIs.

An age-related white matter change scale (Wahlund et al., 2001) with a single-blind method was used to quantitatively evaluate lacunar infarcts and white matter hyperintensity (WMH) based on FLAIR recovery images, and subjects with a rating of >2 were excluded. Five subjects were excluded from the final statistical analysis; two subjects were excluded for excessive motion, and three subjects (one with T2DM and two HCs) were excluded for a WMH score > 2.

### Statistical Analysis

SPSS 18.0 software was used to conduct statistical analysis. Between-group differences in demographics and clinical characteristics were conducted using independent two-sample *t*-tests for normally distributed variables. Non-normally distributed data were evaluated using the Mann–Whitney *U* test. The *χ*^2^ test was used to detect differences in sex and smoking status. A *P* value of <0.05 was considered statistically significant.

The FC analysis was conducted using the DPABI software. Two independent-samples *t-*tests were performed on the FC results of the two groups to identify brain regions with significant differences in connectivity in ROIs after controlling for age. The statistical threshold was set at *P* < 0.001 with a minimum cluster size of 41 voxels within the gray matter mask, and *P* < 0.05 was considered statistically significant (Gaussian random field correction).

The mean FC values of significantly different brain regions between the two groups were extracted. The partial correlations between the mean FC value and clinical/cognitive variables were calculated to investigate the relationship between altered FC in ROIs and cognitive performance, by controlling for age.

## Results

### Clinical and Neuropsychological Data

A total of 57 patients with T2DM and 55 HCs were enrolled in the study. Demographic, clinical, and cognitive data for the two groups are shown in Table 1. No significant differences in age, sex, smoking status, BMI, total cholesterol, triglycerides, low-density lipoprotein, blood pressure, and MMSE and TMT-A scores were observed between the two groups (*P* > 0.05). However, compared with the control group, the T2DM group had increased FBG and HbA1c levels and a higher BDI score, as well as decreased MoCA and CDT scores (all *P*-values < 0.05). In the T2DM group, there were 25 patients with no complications and 32 patients with complications, including nephropathy, peripheral neuropathy, and retinopathy (Supplementary Table 1). Thirteen subjects received dietary restriction, three subjects received insulin, 31 subjects received oral medication (including metformin, sulfonylureas, and acarbose), and 10 subjects received a combination of insulin and oral medication (Supplementary Table 2). Insulin was administered via subcutaneous injection.

**TABLE 1:** Demographic, clinical, and cognitive data of the T2DM and HC groups.

Variable	T2DM (*n* = 57)	HC (*n* = 55)	*P* value
Age (years)	56.09 ± 6.63	54.09 ± 5.42	0.087
Sex (M/F)	37/20	36/19	0.952^#^
Education (years)	13.70 ± 2.64	14.53 ± 2.77	0.110
Total/smoking	57/37	55/30	0.354^#^
BMI (kg/m^2^)	24.42 ± 2.64	24.13 ± 3.12	0.623
T2DM duration (years)	9.75 ± 5.23	−⁣−	−
FBG (mmol/L)	8.54 ± 2.47	5.12 ± 0.76	< 0.01*
HbA1c (%)	8.60 ± 2.40	5.58 ± 0.50	< 0.01*
Systolic blood pressure (mmHg)	127.05 ± 18.00	122.00 ± 9.97	0.070
Diastolic blood pressure (mmHg)	79.58 ± 12.21	81.22 ± 6.67	0.385
TG (mmol/L)	1.72 ± 0.92	1.79 ± 1.14	0.726
TC (mmol/L)	4.54 ± 1.05	4.88 ± 0.91	0.074
LDL (mmol/L)	2.54 ± 0.76	2.81 ± 0.84	0.081
MMSE	27.92 ± 2.23	28.46 ± 1.61	0.148
MoCA	25.36 ± 2.61	26.67 ± 1.91	0.003*
TMT-A (s)	78.15 ± 27.65	71.88 ± 28.08	0.243
CDT	19.35 ± 8.93	22.52 ± 6.56	0.038*
BDI	0 (0, 23)	0 (0, 5)	0.003⁢*Δ

*Note. Normally distributed variables are presented as mean ± standard deviation, and variables that are non-normallydistributed are presented as median (minimum, maximum). T2DM, type 2 diabetes mellitus; HC, healthy control; BMI, body mass index; FBG, fasting blood glucose; TG, triglyceride; TC, total cholesterol; LDL, low-density lipoprotein; HbA1c, glycated hemoglobin; MMSE, Mini-Mental State Examination; MoCA, Montreal Cognitive Assessment; TMT-A, Trail-Making Test A; CDT, Clock-Drawing Test; and BDI, Beck Depression Inventory.*

^Δ^*P* for the Mann–Whitney *U* test; ^#^*P* for the *χ*^2^ test; and *P < 0.05.

### Between-Group FC Differences in Insular Subregions

Compared with HCs, patients with T2DM showed significantly reduced FC between the dAI and the right inferior frontal gyrus, right superior/middle temporal gyrus, right hippocampus, and right precentral gyrus, after controlling for age (*P* < 0.05). FC between the vAI and the right supramarginal gyrus, as well as between the PI and the right precentral/postcentral gyrus, was also reduced in the T2DM group compared with the control group (all *P*-values < 0.05). We did not identify any subregions in which FC was significantly higher in the T2DM group than in the control group (Table 2 and Figures 1B–D).

**TABLE 2:** Aberrant FC in the T2DM group compared with the HC group.

Seed ROI	Brain region	Peak MNI coordinate			Voxelauto (mm^3^)	BA	*t* value
		*X*	*Y*	*Z*			
B dAI	R inferior frontal gyrus	45	27	12	194	46/44	−5.663
	R superior/middle temporal gyrus	54	−48	9	173	22/13	−4.888
	R hippocampus	27	−24	−6	46	20	−5.068
	R precentral gyrus	42	0	39	77	6	−4.786
B vAI	R supramarginal gyrus	57	−39	30	77	40	−4.951
B PI	R precentral/postcentral gyrus	51	3	33	113	4/6	−4.364

*Note. dAI, dorsal anterior insula; vAI, ventral anterior insula; PI, posterior insula; BA, Brodmann’s area; MNI, Montreal Neurological Institute; dlPFC, dorsolateral prefrontal cortex; B, bilateral; and R, right.*

### Correlation Between FC and Clinical/Cognitive Variables

**FIGURE 2:**
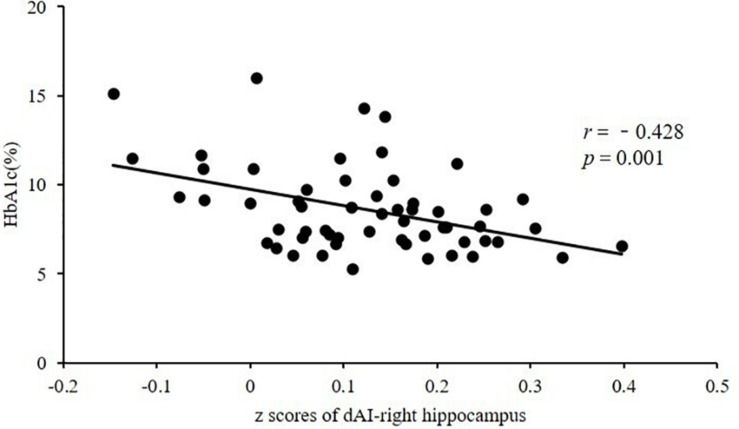
Significant negative correlation between HbA1c and FC in the dAI and right hippocampus in subjects with T2DM (*r* = −0.428, *P* = 0.001). HbA1c, glycated hemoglobin; FC, functional connectivity; dAI, dorsal anterior insula; T2DM, type 2 diabetes mellitus.

After controlling for age, we observed a significant negative correlation between HbA1c and FC in the dAI and right hippocampus in the T2DM group (*r* = −0.428, *P* = 0.001), after Bonferroni correction for *P* (Figure 2). After controlling for age and education, this negative correlation still exists (*r* = −0.431, *P* = 0.001). There were no significant correlations between FC in insular subregions and other clinical/cognitive variables.

## Discussion

This study explored the FC patterns between different insular subregions and the whole brain in patients with T2DM. The results show that compared with the HC group, FC between the dAI and multiple cognitive-related brain regions was reduced, while FC between the vAI and the supramarginal gyrus, as well as between the PI and the precentral/postcentral gyrus, was decreased in patients with T2DM. The distinct FC patterns of insular subregions suggest that functional disconnection of different insular subregions may account for different aspects of T2DM-related brain damage.

### Decreased FC in the dAI in T2DM

The triple-network model suggests that the default-mode network (DMN), central executive network (CEN), and salience network (SN) are the core neural cognitive networks that maintain normal cognitive function (Menon, 2011). FC disorder between these three networks may be the neuropathological basis of many cognitive disorders (Li et al., 2019; Supekar et al., 2019). The dAI is the core node of the SN, which plays a key and causal role in switching/engaging or disengaging between the CEN and DMN (Lowe et al., 2002; Uddin et al., 2011). This study found that brain regions with different dAI FC in patients with T2DM are mainly located in the DMN (hippocampus and superior/middle temporal gyrus) and CEN (inferior frontal gyrus), which may indicate coordination abnormalities among the SN, CEN, and DMN in patients with T2DM. The superior/middle temporal gyrus, the dAI, and the inferior frontal gyrus are involved in higher-level cognitive processes, such as reading comprehension and arithmetic (Fitzhugh et al., 2019; Heidekum et al., 2019; Siok et al., 2020). A recent study (Chang et al., 2018) found strong associations with right anterior insular (AI) connectivity to the right superior/middle temporal gyrus and the inferior frontal gyrus during reading comprehension and arithmetic, which significantly correlated with cognitive function. They consider that the right AI, right superior temporal/middle gyrus, and right inferior frontal gyrus form a cognitive network for reading and arithmetic, and they believe that this network had obvious laterality. Although the precentral gyrus belongs to the sensorimotor cortex, many studies (Sakreida et al., 2013; Wang et al., 2020) have confirmed that it is activated during reading and when performing arithmetic. Our results show that the dAI is abnormally connected to the superior/middle temporal gyrus, inferior frontal gyrus, and precentral gyrus, and they also exhibit laterality, which may imply that T2DM patients have abnormal reading- and arithmetic-related cognitive functions. We analyzed the scores of each cognitive domain of the MoCA scale between groups. The results showed that the arithmetic and sentence repetition domain scores were significantly reduced in patients with T2DM (Supplementary Table 3), which supported our speculation to a certain extent. Although some studies (Messier et al., 2010; Cahana-Amitay et al., 2013) have shown that patients with T2DM have a clear impairment in reading comprehension and arithmetic, as far as we know, there are very few studies that explore the neural mechanism of this impairment. This study may provide some clues for further exploring the neural mechanisms of different higher-level cognitive impairments in patients with T2DM.

The hippocampus is responsible for memory, including working memory and episodic memory (Banquet et al., 2020). Studies show that the hippocampus is mainly involved in retrieval of working memory (Oztekin et al., 2009; Hauser et al., 2020), and the dAI is significantly activated in working memory tasks (Barber et al., 2013) and plays an important role in updating incremental working memory (Halahalli et al., 2015). Working memory is one of the most vulnerable cognitive domains in patients with T2DM (Sadanand et al., 2016). A previous study (Huang et al., 2016) showed that under working memory loads, the activation intensity of the right insula and bilateral hippocampus decreased consistently in patients with T2DM, which confirms that the insula and hippocampus are involved in impaired neural pathways of working memory in patients with T2DM. Therefore, we speculate that decreased connectivity between the dAI and the hippocampus may be related to impairments in working memory. In this study, the CDT score of subjects with T2DM was significantly lower compared with that of HCs, which also indicates that patients with T2DM have abnormal visual memory function. In addition, decreased FC between the dAI and hippocampus was negatively correlated with the HbA1c level, suggesting that chronic high glucose may damage the dAI and the hippocampal pathway. Animal research (Ferreiro et al., 2020) has confirmed that chronic hyperglycemia can damage hippocampal neurons and memory function, and another study showed that high glucose is associated with abnormal memory in patients with T2DM (Zhang et al., 2015). Therefore, it is plausible to speculate that hyperglycemia may affect memory function by disrupting the dAI and the hippocampal pathway.

### Decreased FC in the vAI in T2DM

The vAI is a key region of the emotional SN that integrates external stimuli with internal states to guide behavior (Craig, 2002). The supramarginal gyrus is involved in emotional responses and is more highly activated during negative emotions (Aas et al., 2017). One study (Liu et al., 2015) found that compared with HCs, patients with partially remitted depression showed increased amplitude of low-frequency fluctuation (ALFF) in the left vAI and bilateral supramarginal gyrus. The ALFF value in the right supramarginal gyrus was negatively correlated with Hamilton Depression Rating Scale scores. In addition, patients with bipolar depression showed a disconnection in FC between the vAI and the supramarginal gyrus (Yu et al., 2020). These studies suggest that disconnection between the vAI and supramarginal gyrus is closely related to depression. Several studies (Mezuk et al., 2008; O’Connor et al., 2009; Stuart and Baune, 2012) suggest that the relationship between depression and T2DM is bidirectional or comorbid. In this study, the BDI scores of subjects with T2DM were significantly higher than those of HCs, which suggests that patients with T2DM have a tendency to develop depression. Therefore, we speculate that decreased FC between the vAI and the supramarginal gyrus may be related to the predisposition of patients with T2DM to depression.

### Decreased FC in the PI in T2DM

The PI is involved in sensorimotor processing (Chang et al., 2013), and the precentral gyrus is part of the sensorimotor network. This study found that FC between the PI and precentral gyrus decreased in patients with T2DM, which may indicate sensorimotor abnormalities. Approximately 60% of patients with T2DM have different degrees of peripheral neuropathy (Abbott et al., 2011). Peripheral neuropathy often slows sensorimotor nerve conduction velocity in the distal hand and foot, leading to symptoms such as paresthesia and pain. Previous studies (Zhang et al., 2019; Roy et al., 2020) have confirmed that the sensorimotor cortex (precentral gyrus, postcentral gyrus, and supplementary motor area) of patients with diabetic neuropathy has reduced gray matter volume and abnormal neuronal activity, suggesting that diabetic neuropathy is closely associated with abnormal central sensorimotor function. In this study, we selected HCs matching the demographic data of T2DM patients with peripheral neuropathy (*n* = 18) for between-group analysis; T2DM patients with peripheral neuropathy showed significantly reduced FC between the PI and the right precentral/postcentral gyrus and right inferior parietal lobule (Supplementary Figure 1). This may further confirm that the abnormal FC between PI and the precentral/postcentral gyrus in this study is related to peripheral neuropathy in patients with T2DM. However, it is difficult to determine whether the patients had central sensorimotor abnormalities before peripheral neuropathy occurred. Therefore, future research should investigate whether the sensorimotor cortex in patients with T2DM without peripheral neuropathy is abnormal, which will help to reveal the relationship between T2DM-induced peripheral neuropathy and central sensorimotor cortex damage.

### Limitations

This research has some limitations. First, most subjects with T2DM in this study were revisited patients with a long disease duration, so the results may not be applicable to patients with early T2DM. Second, the treatment plan adopted by patients with T2DM in this study was not consistent; different drugs may bias the study results, but this may be difficult to avoid. Third, although the scores of multiple cognitive domains such as arithmetic and memory in the MoCA scale were significantly reduced in patients with T2DM, the performance evaluation was insufficient. In the future, we will add comprehensive and effective cognitive scales to further explore higher-level cognitive impairment in patients with T2DM. Fourth, although patients with T2DM are often accompanied by depression (Stuart and Baune, 2012), the results might be influenced in the four T2DM patients with depressive symptoms. In future studies, we will investigate whether there is FC disorder in the emotion-related brain regions of T2DM patients without depressive symptoms.

## Conclusion

This study found that different insular subregions present distinct FC patterns with functional brain regions and that abnormal FC in these insular subregions may affect cognitive, emotional, and sensorimotor functions in patients with T2DM. These findings provide more detailed imaging evidence to explore the neural mechanism of the insula in T2DM-related brain damage.

## Data Availability Statement

The original contributions presented in the study are includedin the article/Supplementary Material, further inquiries can bedirected to the corresponding author.

## Ethics Statement

The study was reviewed and approved by The Ethics Committee of Shaanxi Provincial People’s Hospital. The patients/participants provided their written informed consent to participate in this study.

## Author Contributions

DZ drafted the manuscript and designed the experiment. MW performed the statistical analysis. JG contributed to performing the experiments and revised the manuscript. FQ, YL, XY, and YS collected the data. YH, KA, and MC provided technical support. XL contributed to the manuscript review and critique. XZ made contributions to the design of the experiment and revised the manuscript. All authors read and approved the final manuscript.

## Conflict of Interest

The authors declare that the research was conducted in the absence of any commercial or financial relationships that could be construed as a potential conflict of interest.

## Abbreviations

AD: Alzheimer’s disease
BMI: body mass index
CDT: Clock-Drawing Test
CEN: central executive network
dAI: dorsal anterior insula
DMN: default-mode network
FBG: fasting blood glucose
FC: functional connectivity
fMRI: function magnetic resonance imaging
HbA1c: glycated hemoglobin
HC: healthy controls
LDL: low-density lipoprotein cholesterol
MMSE: Mini-Mental State Examination
MoCA: Montreal Cognitive Assessment
PI: posterior insula
SN: salience network
T2DM: type 2 diabetes mellitus
TC: total cholesterol
TG: triglyceride
TMT-A: Trail-Making Test A
vAI: ventral anterior insula.
