# Supplementary figures and images for: Altered Functional Connectivity of Insular Subregions in Type 2 Diabetes Mellitus
Source: Front Neurosci. 2021 Jun 16;15:676624. doi: 10.3389/fnins.2021.676624 (PMC8242202; doi:10.3389/fnins.2021.676624)

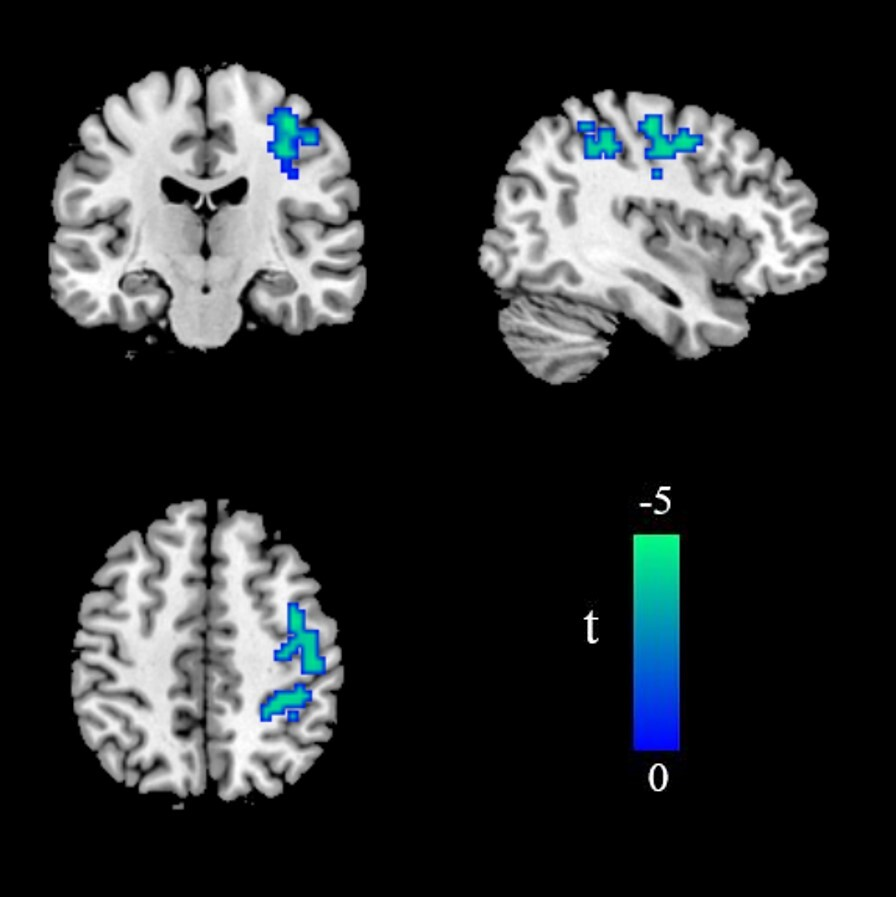

Supplement: Supplementary file 1 [file Image_1.TIF]
