# Supplementary material for: Altered Functional Connectivity of Insular Subregions in Type 2 Diabetes Mellitus
Source: Front Neurosci. 2021 Jun 16;15:676624. doi: 10.3389/fnins.2021.676624 (PMC8242202; doi:10.3389/fnins.2021.676624)
Supplement: Supplementary file 2 [file Table_1.DOCX]

***Supplementary Material***

**Supplementary Table 1.** T2DM complications.

| Complication | Number of patients |
| --- | --- |
| No complications | 25 |
| Nephropathy | 6 |
| Peripheral neuropathy | 11 |
| Retinopathy | 5 |
| Nephropathy + peripheral neuropathy | 5 |
| Nephropathy + retinopathy | 3 |
| Nephropathy + peripheral neuropathy + retinopathy | 2 |

T2DM: type 2 diabetes mellitus.
