# Supplementary material for: Altered Functional Connectivity of Insular Subregions in Type 2 Diabetes Mellitus
Source: Front Neurosci. 2021 Jun 16;15:676624. doi: 10.3389/fnins.2021.676624 (PMC8242202; doi:10.3389/fnins.2021.676624)
Supplement: Supplementary file 3 [file Table_2.DOCX]

***Supplementary Material***

**Supplementary Table 2.** T2DM therapeutic agents.

| Therapeutic agent | Medication | Number of patients |
| --- | --- | --- |
| Dietary restriction |  | 13 |
| Insulin |  | 3 |
|  | Metformin | 16 |
| Oral medication | Metformin + sulfonylureas | 8 |
|  | Metformin + acarbose | 5 |
|  | Sulfonylureas | 2 |
|  | Metformin | 6 |
| Insulin + oral medication | Acarbose | 1 |
|  | Metformin + sulfonylureas | 3 |

T2DM: type 2 diabetes mellitus.
