# Supplementary material for: Altered Functional Connectivity of Insular Subregions in Type 2 Diabetes Mellitus
Source: Front Neurosci. 2021 Jun 16;15:676624. doi: 10.3389/fnins.2021.676624 (PMC8242202; doi:10.3389/fnins.2021.676624)
Supplement: Supplementary file 4 [file Table_3.DOCX]

***Supplementary Material***

**Supplementary Table 3.** The scores of each cognitive domain of the MoCA scale between groups.

| domain | T2DM (*n* = 57) | HC (*n* = 55) | *Z* value | *P* value |
| --- | --- | --- | --- | --- |
| Trail making test | 1 (0, 1) | 1 (0, 1) | -1.193 | 0.233 |
| Copy cube | 1 (0, 1) | 1 (0, 1) | -3.193 | 0.001^*^ |
| [Clock drawing test](javascript:;) | 2 (0, 3) | 2 (1, 3) | -1.400 | 0.162 |
| Naming | 3 (1, 3) | 3 (2, 5) | -2.080 | 0.038^*^ |
| Attention | 3 (0, 3) | 3 (1, 3) | -0.272 | 0.786 |
| Arithmetic | 3 (1, 3) | 3 (1, 3) | -2.456 | 0.014^*^ |
| Sentence repetition | 2 (0, 2) | 2 (0, 2) | -2.218 | 0.027^*^ |
| Verbal fluency test | 1 (1, 1) | 1 (1, 1) | 0.000 | 1.000 |
| [Abstraction](javascript:;) | 2 (0, 2) | 2 (0, 2) | -1.000 | 0.317 |
| Delayed recall | 3 (0, 5) | 4 (0, 5) | -2.591 | 0.010^*^ |
| [Orientation](javascript:;) | 6 (3, 6) | 6 (4, 6) | -0.930 | 0.352 |

Note: Non-normally variables distributed are presented as median (minimum, maximum) and evaluated using the Mann–Whitney U test. T2DM: type 2 diabetes mellitus; HC: healthy control; * *P* < 0.05.
